# Molecular epidemiology and characterization of an outbreak causing *Klebsiella pneumoniae* clone carrying chromosomally located *bla*_CTX-M-15_ at a German University-Hospital

**DOI:** 10.1186/s12866-015-0460-2

**Published:** 2015-06-17

**Authors:** Stephen E. Mshana, Moritz Fritzenwanker, Linda Falgenhauer, Eugen Domann, Torsten Hain, Trinad Chakraborty, Can Imirzalioglu

**Affiliations:** Catholic University of Health and Allied Sciences, Weill Bugando School of Medicine Box, 1464 Mwanza, Tanzania; Institute of Medical Microbiology, Justus-Liebig University, , Schubertstrasse 81, 35392 Giessen, Germany; German Center for Infection Research (DZIF), Partner site Giessen-Marburg-Langen, Campus Giessen, Schubertstrasse 81, 35392 Giessen, Germany

**Keywords:** Hospital, Nosocomial infection, ESBL, Multi-resistance, *Klebsiella*, Chromosomal insertion

## Abstract

**Background:**

Multi-drug resistant *Klebsiella pneumoniae* strains are a common cause of health care associated infections worldwide. Clonal spread of *Klebsiella pneumoniae* isolates carrying plasmid mediated CTX-M-15 have been commonly reported. Limited data is available regarding dissemination of chromosomally encoded CTX-M-15 in *Klebsiella pneumoniae* worldwide.

**Results:**

We examined 23 non-repetitive ESBL-producing *Klebsiella pneumoniae* strains isolated from clinical specimens over a period of 4 months in a German University Hospital. All isolates were characterized to determine their genetic relatedness using Pulsed-Field Gel Electrophoresis (PFGE) and Multi Locus Sequence Typing (MLST). PFGE revealed three clusters (B1, B2, and B3) with a sub-cluster (A3) comprising of 10 isolates with an identical PFGE pattern. All strains of the cluster B3 with similar PFGE patterns were typed as ST101, indicating an outbreak situation. The ESBL allele *bla*_CTX-M-15_ was identified in 16 (69.6 %) of all isolates, including all of the outbreak strains. Within the A3 sub-cluster, the CTX-M-15 allele could not be transferred by conjugation. DNA hybridization studies suggested a chromosomal location of *bla*_CTX-M-15_. Whole genome sequencing located CTX-M-15 within a complete IS*Ecp*-1 transposition unit inserted into an ORF encoding for a putative membrane protein. PCR-based analysis of the flanking regions demonstrated that insertion into this region is unique and present in all outbreak isolates.

**Conclusion:**

This is the first characterization of a chromosomal insertion of *bla*_CTX-M-15_ in *Klebsiella pneumonia* ST101, a finding suggesting that in *Enterobacteriaceae*, chromosomal locations may also act as reservoirs for the spread of *bla*_CTX-M-15_ encoding transposition units.

## Background

*Klebsiella pneumoniae (K. pneumoniae)* is among the most common multi-resistant bacteria causing healthcare associated infections [1–3]. Extended-spectrum β-lactamase (ESBL) producing *K. pneumoniae* are associated with both hospital and community infections [1, 4]. Worldwide, there is an increasing number of reports on CTX-M producing *K. pneumonia* isolates, as evidenced from data presented in different multi-centre studies*. K. pneumoniae* isolates with plasmids harbouring CTX-M-15 have been reported from clinical isolates both from Europe and America [1, 2, 5–7]. The mobility of genetic elements, in particular those conferring antibiotic resistance traits, together with clonal expansion contributes to the persistence of these strains in hospitals and in the community [4, 8]. There are currently only a few reports of chromosomally encoded CTX-M alleles in *Escherichia coli*, *K. pneumoniae, Salmonella enterica* and *Proteus mirabilis* [8–10]. Recently, *K. pneumoniae* strains with chromosomally integrated CTX-M-15 at an undetermined locus were typed as ST1 in a Spanish study [8].

Here we report the clonal outbreak of ESBL producing *K. pneumoniae* carrying *bla*_CTX-M-15_ in a distinct chromosomal location.

## Methods

### Bacterial isolates

Twenty-three non-repetitive, phenotypically proven ESBL producing- *K. pneumoniae* clinical isolates collected consecutively were studied. They represent 11.6 % of all *K. pneumoniae* isolated during the study period. These isolates were taken from miscellaneous specimens including urine, sputum, blood and various swabs over a period of 16 weeks from January 2007 to May 2007. The ESBL phenotype was detected using disk diffusion methods [11]. In addition to routine antimicrobial susceptibility testing by disk diffusion, the Minimal Inhibitory Concentrations (MIC) for cefepime and tigecycline were determined using E-Test stripes (AB BIODISK, Sweden) following the manufacturer’s instruction. Isolates with a MIC of ≥ 8 mg/L for cefepime and a MIC of ≥ 2 mg/L for tigecycline were considered resistant according to recommendations made by the Clinical Laboratory Standards Institute 2010 (CLSI, USA).

### Genetic relatedness and genes encoding for β-lactamases

All isolates were screened for the presence of CTX-M, TEM and SHV genes using primers and methods described previously [12]. PCR fragments were sequenced with the ABI Prism 3100 sequencer (Life technology/Applied Biosystems, USA). DNA sequence analysis was performed using the DNASTAR software (DNASTAR, USA) and homology searches performed using the NCBI Blast database (http://blast.ncbi.nlm.nih.gov/Blast.cgi).

PFGE was performed according to the Pulse Net protocol of the Centre for Disease Control and Prevention (Atlanta, USA). Strain differentiation by PFGE analysis was achieved by comparison of band patterns using Gelcompar II (Applied Maths, Belgium). Patterns were normalized using the molecular weight marker (PFGE Lambda Marker, New England Biolabs, Germany). The similarity coefficient (SAB) of sample pairs was calculated based on band positions by using the DICE metric [13, 14]. Dendograms were generated to visualize relationships among the isolates. The cut-off value in the dendograms was calculated at a SAB of 0.97 as a threshold for defining clusters of genetically similar isolates.

Phylogenetic grouping was performed using a rapid method combining *gyr*A PCR- restricted fragment length polymorphisms analysis (RFLP), *par*C-PCR and adonitol fermentation as described previously [15]. PCR based replicon typing was done as described by Carattoli *et al*. [16] to determine the replicons of plasmid in all clinical isolates. To detect sequence type (ST), MLST was performed as described previously [17]. Briefly, PCR for seven housekeeping genes (*rpo*B, *gap*A, *mdh*, *pgi*, *pho*E, *inf*B and *ton*B) was conducted and the products were directly sequenced and analyzed for single loci variants.

### *Location of the* CTX-M-15 *gene*

Conjugation was performed using plate mating experiments as described [18] . Plasmid analysis was done using the method described [19]. The DNA was transferred on a polyvinyl-based membrane using overnight capillary transfer (CUMC Protocol for Southern Blot, New York) and hybridization done using a CTX-M-15 DIG labelled probe (DIG High Prime DNA Labelling and Detection Starter Kit II, Roche, Germany) following manufacturer’s instructions.

The whole genome sequence of chromosomal DNA of isolate number 39 was determined. Chromosomal DNA was isolated from an overnight culture with the Purelink DNeasy kit according to the manufacturer’s instructions (Invitrogen, Germany). The DNA was then sheared to about 290 bp on the Bioruptor sonication system (Diagenode, Belgium). A chromosomal DNA library was built on the ABI library builder, using the Library Builder™ Fragment Core Kit for SOLiD® 4 according to the protocol demonstrated by Life Technologies (Life Technologies, Germany). The library was then sequenced on a 316 Ion Torrent Chip using the Ion PGM 200 Sequencing Kit (Life Technologies, Germany). The Ion Torrent PGM (Life Technologies, Germany) produced 2.8 million reads with an average length of 185 bp, which amounted to 525.33 Mbp of bases, 324.43 Mbp of which were Q20 or higher quality. The reads were assembled using MIRA 3.4 [20], resulting in 650 contigs larger than or equal to 500 bp with an average coverage of 69.9 ×. The N50 contig size was 18,876 bp. The assembly was improved by manually adding PCR reads in Lasergene Seqman software environment (DNASTAR, USA). A CTX-M-15 carrying insertion element was detected on the chromosome. The inverted repeats of th element were found by comparing the sequence to those of Partridge [21] and Fukui *et al*. (accession number *AB701572.1)*. Primers were designed (Tables 1 and 2) to study the regions flanking CTX-M-15.

**Table 1:** Primers used in this study to investigate CTX-M-15 location

Name	Nucleotide-Sequence (5’-3’)
DOW2.55	GCATCCAAGAGATTCTATCG
DOW2.5	CATACACTCCCTTGTACGG
MP4b-F	TCCTCGACGTTCACGTCT
MP-3 F	AGGGTGAAGCTCAGCTTG
ALA2b	ATGGTTAAAAAATCACTGCG
ALA3b	TTTGCGCATACAGCGGCACAC
P2BC	CGCTGATTTAACAGATTCGG
PIAc	GGCGATCCGCGTGATACCAC
P2Dc	CAGCGCTTTTGCCGTCTAAG
MF-2 F	TGACGATGACCGCTTTCT
MF-2R	CGTCGACTACAGCTTTAA

**Table 2:** PCR fragment sizes with defined primer combinations as calculated based on the chromosomal insertion*

Forward Primer	Reverse Primer	Resulting PCR-Fragment length in base pairs (bp)
ALA2b	ALA3b	88
ALA2b	P2Bc	290
ALA2b	P2Dc	941
ALA2b	MP4b-F	1516
MP-3 F	DOW2.55	1156
MP-3 F	DOW2.5	1718
MP-3 F	ALA3b	2078
MP-3 F	P2Bc	2280
MP-3 F	P2Dc	2931
MP-3 F	MP4b-F	3506
P1Ac	P2Dc	410
P1Ac	MP4b-F	985
MF-2 F	MF-2R	3435

*When the insertion is not present at the examined locus, no specific PCR fragments should be able to be generated, except for the last primer pair, which should then give a PCR product of 459 base pairs (bp)

### Ethical approval

The study was approved by the ethics committee of the medical faculty of the Justus-Liebig-University of Giessen (AZ: 95/11). All samples were taken as part of standard care procedures.

## Results

### ESBL alleles, susceptibility and genetic relatedness

**Fig. 1:**
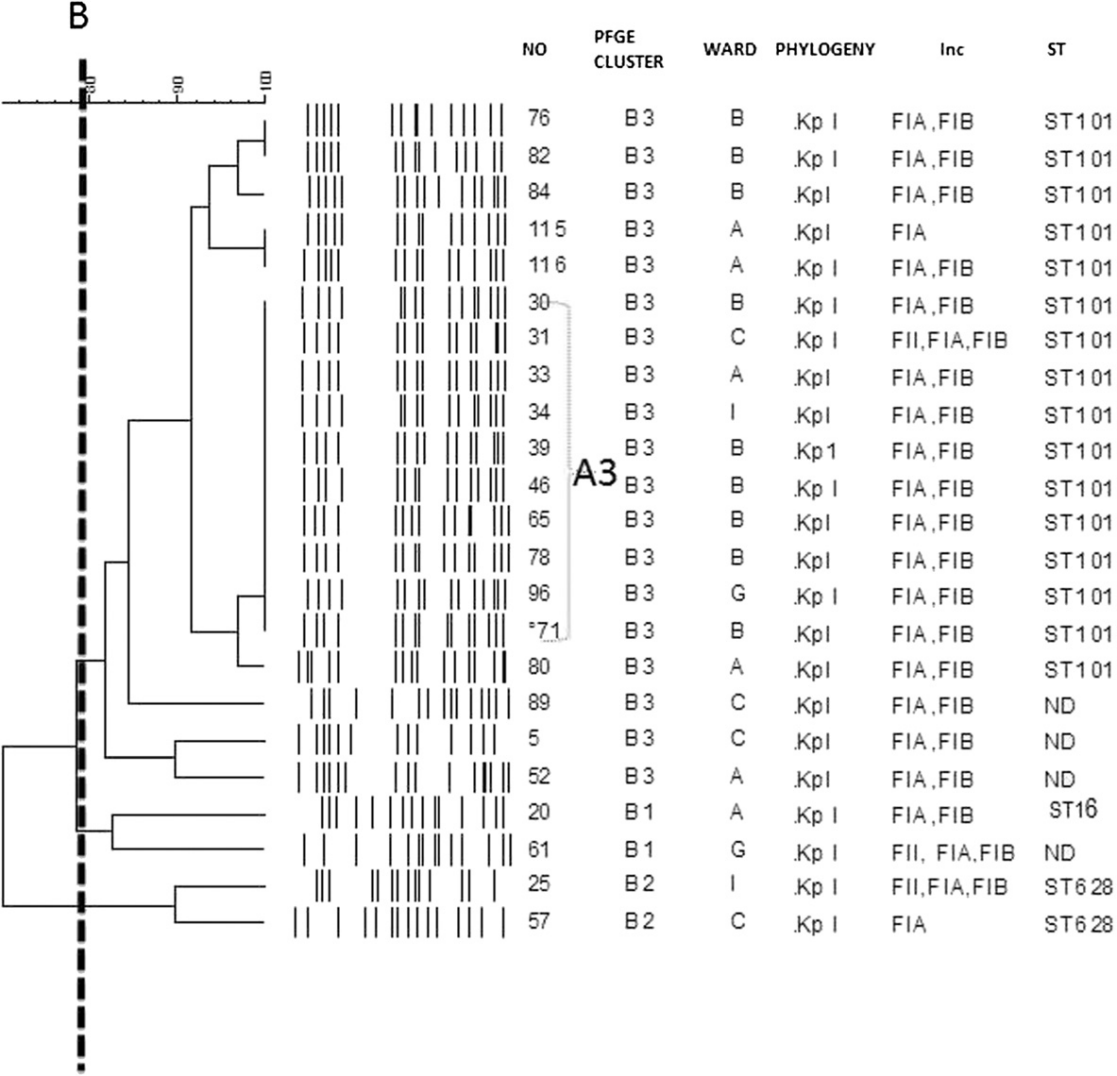
Dendogram (UPGMA, DICE) showing the similarity for the 23 *K. pneumoniae* ESBL positive strains. Line B indicates 80 % similarity. Note the clone A3, Phylogenetic group, wards A-I, PFGE clusters, isolate numbers and sequence type (ST)

PFGE displayed 3 clusters (B1-B3) using a similarity coefficient (SAB) of 0.8 (Fig. 1). Cluster B3 with 19 (83 %) isolates formed the largest group; within this cluster there were 10 isolates which had an identical PFGE pattern (SAB 1.0, sub-cluster A3). Cluster B3 isolates were assigned to sequence type 101 and cluster B2 to the recently reported ST628, while a representative isolate from cluster B1 was assigned to ST16.

All isolates were resistant to cefotaxime, ceftazidime, ceftriaxone, gentamicin, ciprofloxacin, trimethoprim /sulphamethaxazole and cefepime but sensitive to imipenem, meropenem and tigecycline. CTX-M genes were found in 19/23 of *K. pneumoniae* isolates. CTX-M-15 was the most common allele found in 16/23 of isolates (Table 3). Isolate no. 71 had the same PGFE pattern as sub clone A3 isolates and phenotypically confirmed for ESBL production by disk approximation method. Even though the original strain was resistant to cefepime, on subculture it became sensitive with a MIC of 0.125 μg/ml. The *71 isolate was found to lack the CTX-M15 gene.

**Table 3:** Characteristics of the 23 *K. pneumoniae* isolates

Isolate	Ward	PFGE cluster/group	ESBL type	Antibiotic resistance other than β-Lactams	Incompatibility group
20	A	B1	CTX-M-3, TEM-1	GM, TET, SXT, CIP	FIA, FIB
61	G	B1	TEM-104	GM, TET, SXT, CIP	FII, FIA, FIB
25	I	B2	TEM-54	GM, TET, SXT, CIP	FII, FIA, FIB
57	C	B2	CTX-M-15, TEM-1	GM, TET	FIA
76	B	B3	CTX-M-15, TEM-1	GM, TET, SXT, CIP	FIA, FIB
82	B	B3	CTX-M-15, TEM-1	GM, TET, SXT, CIP	FIA, FIB
84	B	B3	CTX-M-15, TEM-1	GM, TET, SXT, CIP	FIA, FIB
115	A	B3	CTX-M-15, TEM-1	GM, TET, SXT, CIP	FIA
116	A	B3	CTX-M-15, TEM-1	GM, TET, SXT, CIP	FIA, FIB
30	B	B3	CTX-M-15, TEM-1	GM, TET, SXT, CIP	FIA, FIB
33	A	B3	CTX-M-15, TEM-1	GM, TET, SXT, CIP	FIA, FIB
34	I	B3	CTX-M-15, TEM-1	GM, TET, SXT, CIP	FIA, FIB
39	B	B3	CTX-M-15, TEM-1	GM, TET, SXT, CIP	FIA, FIB
31	C	B3	CTX-M-15, TEM-1	GM, TET, SXT, CIP	FIA, FIB
46	B	B3	CTX-M-15, TEM-1	GM, TET, SXT, CIP	FIA, FIB
65	B	B3	CTX-M-15, TEM-1	GM, TET, SXT, CIP	FIA, FIB
^*^71	B	B3	TEM-1	GM, TET, SXT, CIP	FIA, FIB
78	B	B3	CTX-M-15, TEM-1	GM, TET, SXT, CIP	FIA, FIB
96	G	B3	CTX-M-15, TEM-1	GM, TET, SXT, CIP	FIA, FIB
80	A	B3	CTX-M-15, TEM-1	GM, TET, SXT, CIP	FIA, FIB
89	C	B3	CTX-M-3, TEM-1	GM, TET, SXT, CIP	FIA, FIB
5	C	B3	TEM-104	GM, TET, SXT, CIP	ND
52	A	B3	CTX-M-3, TEM-1	GM, SXT, CIP	FIA, FIB

*This isolate was positive for ESBL phenotypically but no ESBL gene was found

GM: Gentamicin, TET: Tetracycline, CIP: Ciprofloxacin, SXT: Sulphamethaxazole/trimethoprim, ND: not determined

### Plasmid analysis and chromosomal location

**Fig. 2:**
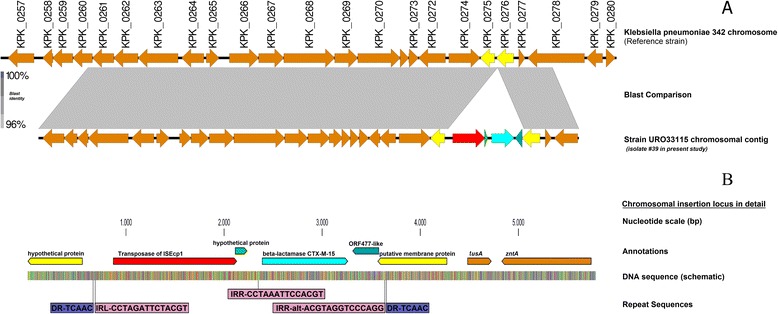
Schematic diagram showing the chromosomal insertion site and the surrounding environment. **a** overview and chromosomal context compared to the reference genome of *K. pneumoniae* 342, accession number CP000964.1 **b** detailed view of the chromosomal insertion locus of bla_CTX-M-15_ in *Klebsiella pneumoniae* UR033115 (investigated clone no. 39) and binding sites of primers as seen in the Tables 1 and 2

Plasmid analysis revealed that all isolates harboured multiple plasmids of various sizes ranging from less than 48.5 kb to 436.5 kb. Despite this fact, the CTX-M-15 resistance gene was not transferable from PFGE B3 sub cluster A3 isolates (n = 10) by conjugation or transformation in multiple attempts made. Hybridization using a CTX-M-15 DIG labelled probe indicated a chromosomal location in five isolates of PFGE type B3 subclone A3. Several colonies selected on a lysogeny broth plate containing 2 mg/L cefotaxime with a positive CTX-M PCR and phenotypically confirmed for ESBL production, were investigated for the insertion locus of the CTX-M-15 allele. In all cases a complete CTX-M-15/IS*Ecp*1-element of 2,971 bp with flanking direct repeats (TCAAC) was found (Fig. 2 a/b). Comparison of the contig sequence obtained by whole-genome sequencing of a representative isolate (no.39/URO33115) of the PFGEsub cluster A3, which harbours the CTX-M-15/IS*Ecp1*-element, with a reference strain sequence (*K. pneumoniae* 342, accession number CP000964.1) revealed that the element is inserted between the coding sequences (CDS) KPK_0275 (hypothetical protein) and KPK_0276 (putative membrane protein, Fig. 2 a/b), indicating integration of the transposition unit harbouring the IS*Ecp*1 insertion sequence and the CTX-M-15 gene into the chromosome of the outbreak strain. The resulting contig sequence was deposited in the GenBank nucleotide database under accession number HG780615.

The other 9 strains within the sub-cluster A3 (ST101) were all tested positive for the same insertion locus using a specifically developed, sequence-based PCR-assay underlining their clonal identity (Table 2).

## Discussion

The study documents the presence of *K. pneumoniae* clones carrying a chromosomally located CTX-M-15 in a large University Hospital in Germany. The high prevalence of CTX-M-15 is consistent with many other studies worldwide [4, 6] but unlike those studies in a subset of isolates, the CTX-M-15 gene was not transferable by conjugation or transformation and DNA hybridization studies indicated a chromosomal localization for the gene. This was confirmed by whole genome sequencing and PCR mapping studies. Descriptions of chromosomal locations of ESBL genes in clinical isolates are extremely rare, with only a single report of chromosomally located CTX-M-15 at an undetermined location for a *K. pneumoniae* ST1 isolate and unlike the outbreak isolates reported here which were typed as ST101 [8]*.* ST101 belongs to the clonal complex 11; this clonal complex of *K. pneumoniae* has been associated with different ESBL alleles including CTX-M-15, OXA-48 and more recently KPC [22, 23]. It is now globally disseminated and is associated with healthcare-associated infections in North America, Europe and Asia [23–25]. It is worth noting that in most of the studies describing the epidemiology of *bla*_CTX-M-15_, the location of the gene is not always stated, thus a chromosomal location may be a more common phenomenon than previously suspected.

In the present study the complete IS*Ecp*1/CTX-M-15 transposition unit with inverted repeats was found to be located on the chromosome, resulting in the duplication of a 5 bp repeat sequence (TCAAC). The organization of this transposition unit was similar as in conjugative IncF plasmids [12, 26] where the CTX-M-15 gene was also found 49 bp downstream of IS*Ecp*1. As described in reference plasmids the right inverted repeat was located in ORF477 (378 bp; GenBank accession no. HQ157357), downstream of the CTX-M-15 gene. This organization has been observed in 98 % of IncF plasmids carrying CTX-M-15 and also in the chromosome of *Kluyvera ascorbata* carrying CTX-M-3 [27]. Chromosomally encoded β-lactamases from some *Kluyvera* species have been proposed as a possible source of CTX-M enzymes. The finding from this study suggests that other *Enterobacteriaceae* might also act as intermediate hosts and as a possible source of CTX-M genes.

## Conclusions

For the first time we describe the site of integration of a complete CTX-M-15/IS*Ecp*1 element into the chromosome of a *K. pneumoniae* ST101 strain. This insertion site is distinct and differs markedly from a recently published sequence of a *K. pneumoniae* ST11 isolate where CTX-M-15 alleles were present both on a plasmid as well as a chromosomal location (CP006659.1, *K. pneumoniae* ATCC BAA-2146) [28]. The site of insertion also differs from that reported for the ST131 *E. coli* JJ1886 strain [29]. Our studies also reveal the presence of several large IncF plasmids in *K. pneumoniae* which have hitherto been poorly characterized, warranting additional studies. Using data derived in this study we have devised a specific PCR-based assay to examine for the chromosomal insertion of CTX-M-15 in ST101 *K. pneumoniae* isolates.

### Availability of supporting data

The data set supporting the results of this article is available in GenBank under the accession number HG780615.

## References

[1] Peirano G, Sang JHK, Pitondo-Silva A, Laupland KB, Pitout JD (2012). Molecular epidemiology of extended-spectrum-β-lactamase-producing Klebsiella pneumoniae over a 10 year period in Calgary, Canada. J Antimicrob Chemother.

[2] Romero L, Lopez L, Rodríguez‐Baño J, Ramón Hernández J, Martínez‐Martínez L, Pascual A (2005). Long‐term study of the frequency of Escherichia coli and Klebsiella pneumoniae isolates producing extended‐spectrum β‐lactamases. Clin Microbiol Infect.

[3] Mshana SE, Hain T, Domann E, Lyamuya EF, Chakraborty T, Imirzalioglu C (2013). Predominance of Klebsiella pneumoniae ST14 carrying CTX-M-15 causing neonatal sepsis in Tanzania. BMC Infect Dis.

[4] Barguigua A, El Otmani F, Talmi M, Bourjilat F, Haouzane F, Zerouali K (2011). Characterization of extended-spectrum β-lactamase-producing Escherichia coli and Klebsiella pneumoniae isolates from the community in Morocco. J Med Microbiol.

[5] Poirel L, Bonnin RA, Nordmann P (2012). Genetic support and diversity of acquired extended-spectrum β-lactamases in Gram-negative rods. Infect Genet Evol.

[6] Younes A, Hamouda A, Dave J, Amyes S (2011). Prevalence of transferable blaCTX-M-15 from hospital-and community-acquired Klebsiella pneumoniae isolates in Scotland. J Antimicrob Chemother.

[7] Coelho A, Mirelis B, Alonso-Tarrés C, Larrosa MN, Miró E, Abad RC (2009). Detection of three stable genetic clones of CTX-M-15-producing Klebsiella pneumoniae in the Barcelona metropolitan area, Spain. J Antimicrob Chemother.

[8] Coelho A, González-López JJ, Miró E, Alonso-Tarrés C, Mirelis B, Larrosa MN (2010). Characterisation of the CTX-M-15-encoding gene in Klebsiella pneumoniae strains from the Barcelona metropolitan area: plasmid diversity and chromosomal integration. Int J Antimicrob Agents.

[9] Fabre L, Delauné A, Espié E, Nygard K, Pardos M, Polomack L (2009). Chromosomal integration of the extended-spectrum β-lactamase gene blaCTX-M-15 in Salmonella enterica serotype Concord isolates from internationally adopted children. Antimicrob Agents Chemother.

[10] Song W, Kim J, Bae IK, Jeong SH, Seo YH, Shin JH (2011). Chromosome-encoded AmpC and CTX-M extended-spectrum β-lactamases in clinical isolates of Proteus mirabilis from Korea. Antimicrob Agents Chemother.

[11] Mshana SE, Imirzalioglu C, Hossain H, Hain T, Domann E, Chakraborty T (2009). Conjugative IncFI plasmids carrying CTX-M-15 among Escherichia coli ESBL producing isolates at a University hospital in Germany. BMC Infect Dis.

[12] Baraniak A, Fiett J, Hryniewicz W, Nordmann P, Gniadkowski M (2002). Ceftazidime-hydrolysing CTX-M-15 extended-spectrum β-lactamase (ESBL) in Poland. J Antimicrob Chemother.

[13] Hunter PR, Fraser C (1989). Application of a numerical index of discriminatory power to a comparison of four physiochemical typing methods for Candida albicans. J Clin Microbiol.

[14] Hunter PR, Gaston MA (1988). Numerical index of the discriminatory ability of typing systems: an application of Simpson’s index of diversity. J Clin Microbiol.

[15] Brisse S, Van Himbergen T, Kusters K, Verhoef J (2004). Development of a rapid identification method for Klebsiella pneumoniae phylogenetic groups and analysis of 420 clinical isolates. Clin Microbiol Infect.

[16] Carattoli A, Miriagou V, Bertini A, Loli A, Colinon C, Villa L (2006). Replicon typing of plasmids encoding resistance to newer β-lactams. Emerg Infect Dis.

[17] Diancourt L, Passet V, Verhoef J, Grimont PA, Brisse S (2005). Multilocus sequence typing of Klebsiella pneumoniae nosocomial isolates. J Clin Microbiol.

[18] Schmitt J, Jacobs E, Schmidt H (2007). Molecular characterization of extended-spectrum beta-lactamases in Enterobacteriaceae from patients of two hospitals in Saxony, Germany. J Med Microbiol.

[19] Barton BM, Harding GP, Zuccarelli AJ (1995). A general method for detecting and sizing large plasmids. Anal Biochem.

[20] Chevreux B, Pfisterer T, Drescher B, Driesel AJ, Müller WE, Wetter T (2004). Using the miraEST assembler for reliable and automated mRNA transcript assembly and SNP detection in sequenced ESTs. Genome Res.

[21] Partridge SR (2011). Analysis of antibiotic resistance regions in Gram‐negative bacteria. FEMS Microbiol Rev.

[22] Potron A, Poirel L, Rondinaud E, Nordmann P (2013). Intercontinental spread of OXA-48 beta-lactamase-producing Enterobacteriaceae over a 11-year period, 2001 to 2011. Euro Surveill.

[23] Seki LM, Pereira PS, de Souza Mda P, Conceição MS, Marques EA, Porto CO (2011). Molecular epidemiology of KPC-2-producing Klebsiella pneumoniae isolates in Brazil: the predominance of sequence type 437. Diagn Microbiol Infect Dis.

[24] Leavitt A, Carmeli Y, Chmelnitsky I, Goren MG, Ofek I, Navon-Venezia S (2010). Molecular epidemiology, sequence types, and plasmid analyses of KPC-producing Klebsiella pneumoniae strains in Israel. Antimicrob Agents Chemother.

[25] Li B, Yi Y, Wang Q, Woo PC, Tan L, Jing H (2012). Analysis of drug resistance determinants in Klebsiella pneumoniae isolates from a tertiary-care hospital in Beijing, China. PloS One.

[26] Boyd DA, Tyler S, Christianson S, McGeer A, Muller MP, Willey BM (2004). Complete nucleotide sequence of a 92-kilobase plasmid harboring the CTX-M-15 extended-spectrum beta-lactamase involved in an outbreak in long-term-care facilities in Toronto, Canada. Antimicrob Agents Chemother.

[27] Rodríguez MM, Power P, Radice M, Vay C, Famiglietti A, Galleni M (2004). Chromosome-encoded CTX-M-3 from Kluyvera ascorbata: a possible origin of plasmid-borne CTX-M-1-derived cefotaximases. Antimicrob Agents Chemother.

[28] Hudson CM, Bent ZW, Meagher RJ, Williams KP (2014). Resistance Determinants and Mobile Genetic Elements of an NDM-1-Encoding Klebsiella pneumoniae Strain. PLoS One.

[29] Andersen PS, Stegger M, Aziz M, Contente-Cuomo T, Gibbons HS, Keim P (2013). Complete genome sequence of the epidemic and highly virulent CTX-M-15-producing H30-Rx subclone of Escherichia coli ST131. Genome Announc.

